# 5,5′-[Methyl­enebis(sulfanedi­yl)]bis­(1,3,4-thia­diazol-2-amine)

**DOI:** 10.1107/S1600536808030122

**Published:** 2008-09-24

**Authors:** Fankun Meng

**Affiliations:** a Department of Chemistry and Chemical Engineering, Daqing Normal University, 163712 Daqing, Heilongjiang, People’s Republic of China

## Abstract

In the crystal structure of the title compound, C_5_H_6_N_6_S_4_, the mol­ecules are linked by strong N—H⋯N hydrogen bonds into a two-dimensional network and an intra­molecular C—H⋯S inter­action also occurs.

## Related literature

For the multiple coordination environment of this ligand, see: Ma *et al.* (2007 ▶).
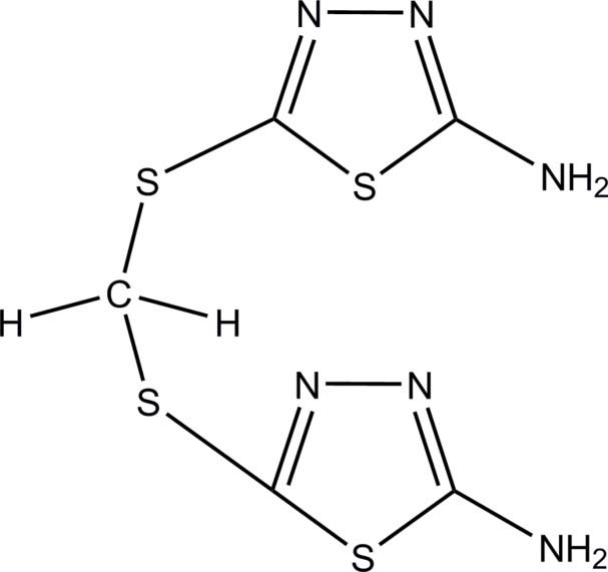


## Experimental

### 

#### Crystal data

C_5_H_6_N_6_S_4_
*M* *_r_* = 278.40
Triclinic, 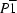

*a* = 5.457 (3) Å
*b* = 7.316 (4) Å
*c* = 13.623 (8) Å
α = 81.746 (8)°
β = 88.864 (8)°
γ = 74.858 (8)°
*V* = 519.5 (5) Å^3^
*Z* = 2
Mo *K*α radiation
μ = 0.89 mm^−1^
*T* = 298 (2) K
0.28 × 0.19 × 0.14 mm

#### Data collection

Siemens SMART CCD area-detector diffractometer
Absorption correction: multi-scan (*SADABS*; Sheldrick, 1996 ▶) *T*_min_ = 0.789, *T*_max_ = 0.886
2686 measured reflections
1801 independent reflections
1525 reflections with *I* > 2σ(*I*)
*R*_int_ = 0.034

#### Refinement

*R*[*F* ^2^ > 2σ(*F* ^2^)] = 0.047
*wR*(*F* ^2^) = 0.130
*S* = 1.00
1801 reflections
136 parameters
H-atom parameters constrained
Δρ_max_ = 0.47 e Å^−3^
Δρ_min_ = −0.65 e Å^−3^

### 

Data collection: *SMART* (Siemens, 1996 ▶); cell refinement: *SAINT* (Siemens, 1996 ▶); data reduction: *SAINT*; program(s) used to solve structure: *SHELXS97* (Sheldrick, 2008 ▶); program(s) used to refine structure: *SHELXL97* (Sheldrick, 2008 ▶); molecular graphics: *SHELXTL* (Sheldrick, 2008 ▶); software used to prepare material for publication: *SHELXTL*.

## Supplementary Material

Crystal structure: contains datablocks I, global. DOI: 10.1107/S1600536808030122/bx2173sup1.cif

Structure factors: contains datablocks I. DOI: 10.1107/S1600536808030122/bx2173Isup2.hkl

Additional supplementary materials:  crystallographic information; 3D view; checkCIF report

## Figures and Tables

**Table 1:** Hydrogen-bond geometry (Å, °)

*D*—H⋯*A*	*D*—H	H⋯*A*	*D*⋯*A*	*D*—H⋯*A*
N3—H3*A*⋯N5^i^	0.86	2.18	2.999 (4)	158
N6—H6*A*⋯N2^i^	0.86	2.18	3.023 (4)	168
N6—H6*B*⋯N1^ii^	0.86	2.19	3.021 (4)	162
C5—H5*A*⋯S1	0.97	2.82	3.364 (4)	116

Symmetry codes: (i) 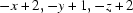; (ii) 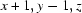.

**Fig. 1.**
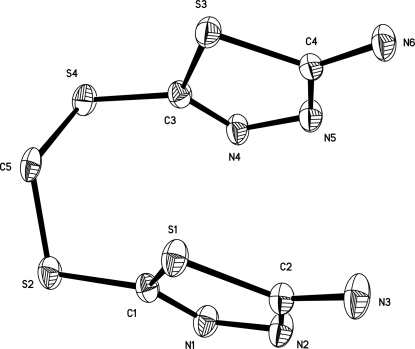
The structure of the title complex, showing 30% probability displacement ellipsoids and the atom-numbering scheme. The H atoms are omitted for clarity.

**Fig. 2.**
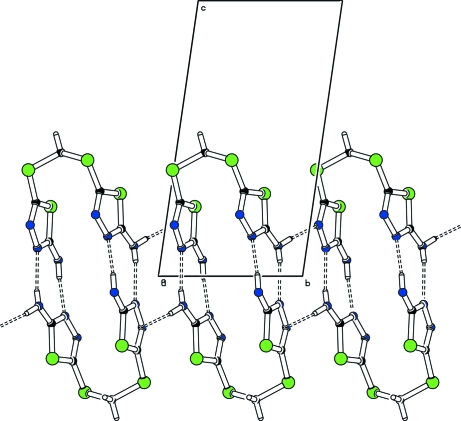
The crystal packing of (I), viewed along the a axis. Dashed lines show intermolecular hydrogen bonds.

## supplementary crystallographic information

### Comment

5-amino-4*H*-pyrazole-3-thiol ligand and its derivatives are widely studied because of their multiply coordination environment (Ma, *et al.*, 2007). They represent a class of highly useful compounds in which the presence of S and N atoms renders various hydrogen bonding motifs leading to the formation of versatile supramolecular architecture. As continuous study of this ligand we report here the structure of the title compound,(I)(Fig. 1). In the crystal structure of the title compound, the molecules are linked by strong N—H···N hydrogen bonds into a two-dimensional network, Fig 2. An intramolecular C-H···S interaction also occurs.

### Experimental

5-amino-1,3,4-thiadiazole-2-thiol(2 mmol), and sodium ethanolate were dissolved in  ethanol, and the mixture was stirred for 4 h at 323 K. After cooling at room temperature, the solution was filtered. The solvent was removed from the filtrate under vacuum, and the solid residue was recrystallized from diethylether; colorless crystals suitable for X-Ray diffraction study were obtained. Yield, 81%. m.p. 368 K. Analysis, calculated for C_5_H_6_N_6_S_4_: C 21.57, H 2.17, N 30.19; found: C 21.36, H 2.43, N 30.32. The elemental analyses were performed with a Perkine Elemer PE2400II instrument.

### Refinement

The amido H atoms were placed in idealized positions and constrained to ride on their parent atoms, with amido N—H = 0.86 Å. The *U*_iso_(H) values were set at 1.2*U*_eq_(N) for the amido H atoms. The methylene H atoms could be located in difference Fourier maps. It was refined with distance restraints of C–H = 0.97 Å and U_iso_(H)= 1.2U_eq_(C).

### Crystal data

**Table:**

C_5_H_6_N_6_S_4_	*Z* = 2
*M**_r_* = 278.40	*F*(000) = 284
Triclinic, *P*1	*D*_x_ = 1.780 Mg m^−^^3^
Hall symbol: -P 1	Mo *K*α radiation, λ = 0.71073 Å
*a* = 5.457 (3) Å	Cell parameters from 1816 reflections
*b* = 7.316 (4) Å	θ = 2.9–28.3°
*c* = 13.623 (8) Å	µ = 0.89 mm^−^^1^
α = 81.746 (8)°	*T* = 298 K
β = 88.864 (8)°	Block, colourless
γ = 74.858 (8)°	0.28 × 0.19 × 0.14 mm
*V* = 519.5 (5) Å^3^	

### Data collection

**Table:**

Siemens SMART CCD area-detector diffractometer	1801 independent reflections
Radiation source: fine-focus sealed tube	1525 reflections with *I* > 2σ(*I*)
graphite	*R*_int_ = 0.034
φ and ω scans	θ_max_ = 25.0°, θ_min_ = 2.9°
Absorption correction: multi-scan (SADABS; Sheldrick, 1996)	*h* = −5→6
*T*_min_ = 0.789, *T*_max_ = 0.886	*k* = −7→8
2686 measured reflections	*l* = −15→16

### Refinement

**Table:**

Refinement on *F*^2^	Primary atom site location: structure-invariant direct methods
Least-squares matrix: full	Secondary atom site location: difference Fourier map
*R*[*F*^2^ > 2σ(*F*^2^)] = 0.047	Hydrogen site location: inferred from neighbouring sites
*wR*(*F*^2^) = 0.130	H-atom parameters constrained
*S* = 1.00	*w* = 1/[σ^2^(*F*_o_^2^) + (0.09*P*)^2^ + 0.0868*P*] where *P* = (*F*_o_^2^ + 2*F*_c_^2^)/3
1801 reflections	(Δ/σ)_max_ < 0.001
136 parameters	Δρ_max_ = 0.47 e Å^−^^3^
0 restraints	Δρ_min_ = −0.65 e Å^−^^3^

### Special details

**Table:**

Geometry. All e.s.d.'s (except the e.s.d. in the dihedral angle between two l.s. planes) are estimated using the full covariance matrix. The cell e.s.d.'s are taken into account individually in the estimation of e.s.d.'s in distances, angles and torsion angles; correlations between e.s.d.'s in cell parameters are only used when they are defined by crystal symmetry. An approximate (isotropic) treatment of cell e.s.d.'s is used for estimating e.s.d.'s involving l.s. planes.
Refinement. Refinement of *F*^2^ against ALL reflections. The weighted *R*-factor *wR* and goodness of fit *S* are based on *F*^2^, conventional *R*-factors *R* are based on *F*, with *F* set to zero for negative *F*^2^. The threshold expression of *F*^2^ > σ(*F*^2^) is used only for calculating *R*-factors(gt) *etc*. and is not relevant to the choice of reflections for refinement. *R*-factors based on *F*^2^ are statistically about twice as large as those based on *F*, and *R*- factors based on ALL data will be even larger.

### Fractional atomic coordinates and isotropic or equivalent isotropic displacement parameters (Å^2^)

**Table:**

	*x*	*y*	*z*	*U*_iso_*/*U*_eq_	
N1	0.7436 (5)	0.9404 (4)	0.81430 (19)	0.0378 (6)	
N2	0.8979 (5)	0.8670 (4)	0.89745 (18)	0.0391 (6)	
N3	1.3224 (5)	0.7099 (4)	0.94128 (19)	0.0466 (7)	
H3A	1.2912	0.7013	1.0037	0.056*	
H3B	1.4740	0.6646	0.9216	0.056*	
N4	0.7147 (5)	0.4974 (4)	0.77661 (18)	0.0393 (6)	
N5	0.8645 (5)	0.3914 (4)	0.85485 (18)	0.0404 (6)	
N6	1.2775 (5)	0.1997 (4)	0.89166 (18)	0.0417 (7)	
H6A	1.2482	0.1837	0.9541	0.050*	
H6B	1.4246	0.1472	0.8701	0.050*	
S1	1.18916 (14)	0.81717 (11)	0.74813 (5)	0.0366 (3)	
S2	0.72542 (15)	1.00787 (11)	0.61469 (6)	0.0398 (3)	
S3	1.13900 (15)	0.34844 (11)	0.70097 (5)	0.0378 (3)	
S4	0.68122 (15)	0.60763 (11)	0.57878 (5)	0.0403 (3)	
C1	0.8660 (5)	0.9238 (4)	0.7323 (2)	0.0311 (6)	
C2	1.1362 (6)	0.7931 (4)	0.8752 (2)	0.0336 (7)	
C3	0.8273 (6)	0.4904 (4)	0.6922 (2)	0.0329 (7)	
C4	1.0953 (5)	0.3057 (4)	0.8283 (2)	0.0307 (6)	
C5	0.8374 (6)	0.7999 (4)	0.55130 (19)	0.0375 (7)	
H5A	1.0170	0.7468	0.5668	0.045*	
H5B	0.8213	0.8433	0.4805	0.045*	

### Atomic displacement parameters (Å^2^)

**Table:**

	*U*^11^	*U*^22^	*U*^33^	*U*^12^	*U*^13^	*U*^23^
N1	0.0323 (14)	0.0464 (15)	0.0301 (13)	−0.0032 (11)	−0.0026 (11)	−0.0024 (11)
N2	0.0327 (14)	0.0533 (15)	0.0252 (12)	−0.0018 (11)	−0.0026 (10)	−0.0018 (11)
N3	0.0344 (15)	0.0664 (18)	0.0280 (13)	0.0011 (13)	−0.0066 (11)	0.0039 (13)
N4	0.0341 (14)	0.0451 (14)	0.0297 (13)	0.0008 (11)	−0.0016 (11)	0.0037 (11)
N5	0.0353 (15)	0.0494 (15)	0.0286 (13)	−0.0012 (12)	−0.0006 (11)	0.0020 (11)
N6	0.0337 (14)	0.0516 (16)	0.0293 (13)	0.0020 (12)	−0.0038 (11)	0.0054 (12)
S1	0.0276 (4)	0.0498 (5)	0.0267 (4)	−0.0028 (3)	−0.0016 (3)	0.0002 (3)
S2	0.0398 (5)	0.0424 (5)	0.0300 (4)	−0.0032 (3)	−0.0097 (3)	0.0062 (3)
S3	0.0356 (5)	0.0438 (5)	0.0276 (4)	−0.0004 (3)	−0.0002 (3)	−0.0024 (3)
S4	0.0401 (5)	0.0507 (5)	0.0287 (4)	−0.0111 (4)	−0.0122 (3)	−0.0014 (3)
C1	0.0298 (15)	0.0323 (14)	0.0285 (14)	−0.0068 (12)	−0.0049 (12)	0.0028 (11)
C2	0.0353 (16)	0.0368 (15)	0.0264 (14)	−0.0075 (12)	−0.0040 (12)	0.0002 (12)
C3	0.0321 (15)	0.0343 (15)	0.0302 (15)	−0.0070 (12)	−0.0056 (12)	−0.0006 (12)
C4	0.0336 (16)	0.0290 (14)	0.0275 (14)	−0.0065 (12)	−0.0031 (12)	−0.0004 (11)
C5	0.0370 (17)	0.0525 (18)	0.0186 (13)	−0.0082 (14)	−0.0049 (12)	0.0032 (12)

### Geometric parameters (Å, °)

**Table:**

N1—C1	1.295 (4)	N6—H6B	0.8600
N1—N2	1.381 (4)	S1—C1	1.736 (3)
N2—C2	1.319 (4)	S1—C2	1.741 (3)
N3—C2	1.330 (4)	S2—C1	1.747 (3)
N3—H3A	0.8600	S2—C5	1.818 (3)
N3—H3B	0.8600	S3—C4	1.741 (3)
N4—C3	1.294 (4)	S3—C3	1.742 (3)
N4—N5	1.368 (3)	S4—C3	1.752 (3)
N5—C4	1.321 (4)	S4—C5	1.819 (3)
N6—C4	1.332 (4)	C5—H5A	0.9700
N6—H6A	0.8600	C5—H5B	0.9700
			
C1—N1—N2	112.9 (2)	S1—C1—S2	121.83 (17)
C2—N2—N1	112.6 (2)	N2—C2—N3	124.8 (3)
C2—N3—H3A	120.0	N2—C2—S1	113.2 (2)
C2—N3—H3B	120.0	N3—C2—S1	122.0 (2)
H3A—N3—H3B	120.0	N4—C3—S3	113.7 (2)
C3—N4—N5	113.4 (2)	N4—C3—S4	123.8 (2)
C4—N5—N4	113.0 (2)	S3—C3—S4	122.55 (17)
C4—N6—H6A	120.0	N5—C4—N6	124.1 (3)
C4—N6—H6B	120.0	N5—C4—S3	112.8 (2)
H6A—N6—H6B	120.0	N6—C4—S3	123.1 (2)
C1—S1—C2	86.94 (14)	S2—C5—S4	117.40 (16)
C1—S2—C5	101.78 (13)	S2—C5—H5A	108.0
C4—S3—C3	87.09 (13)	S4—C5—H5A	108.0
C3—S4—C5	101.31 (13)	S2—C5—H5B	108.0
N1—C1—S1	114.3 (2)	S4—C5—H5B	108.0
N1—C1—S2	123.8 (2)	H5A—C5—H5B	107.2
			
C1—N1—N2—C2	−1.0 (4)	N5—N4—C3—S3	−0.1 (3)
C3—N4—N5—C4	0.9 (4)	N5—N4—C3—S4	178.7 (2)
N2—N1—C1—S1	−0.7 (3)	C4—S3—C3—N4	−0.6 (2)
N2—N1—C1—S2	−178.4 (2)	C4—S3—C3—S4	−179.3 (2)
C2—S1—C1—N1	1.5 (2)	C5—S4—C3—N4	107.8 (3)
C2—S1—C1—S2	179.36 (19)	C5—S4—C3—S3	−73.5 (2)
C5—S2—C1—N1	−130.4 (3)	N4—N5—C4—N6	177.8 (3)
C5—S2—C1—S1	52.0 (2)	N4—N5—C4—S3	−1.4 (3)
N1—N2—C2—N3	−178.6 (3)	C3—S3—C4—N5	1.1 (2)
N1—N2—C2—S1	2.2 (3)	C3—S3—C4—N6	−178.1 (3)
C1—S1—C2—N2	−2.1 (2)	C1—S2—C5—S4	77.73 (18)
C1—S1—C2—N3	178.7 (3)	C3—S4—C5—S2	−79.41 (18)

### Hydrogen-bond geometry (Å, °)

**Table:**

*D*—H···*A*	*D*—H	H···*A*	*D*···*A*	*D*—H···*A*
N3—H3A···N5^i^	0.86	2.18	2.999 (4)	158
N6—H6A···N2^i^	0.86	2.18	3.023 (4)	168
N6—H6B···N1^ii^	0.86	2.19	3.021 (4)	162
C5—H5A···S1	0.97	2.82	3.364 (4)	116

Symmetry codes: (i) −*x*+2, −*y*+1, −*z*+2; (ii) *x*+1, *y*−1, *z*.
